# Current Status and Future Perspectives on Therapeutic Potential of Apigenin: Focus on Metabolic-Syndrome-Dependent Organ Dysfunction

**DOI:** 10.3390/antiox10101643

**Published:** 2021-10-19

**Authors:** Waqas Alam, Carmine Rocca, Haroon Khan, Yaseen Hussain, Michael Aschner, Anna De Bartolo, Nicola Amodio, Tommaso Angelone, Wai San Cheang

**Affiliations:** 1 Department of Pharmacy, Abdul Wali Khan University Mardan, Mardan 23200, Pakistan; waqasalam@awkum.edu.pk; 2 Laboratory of Cellular and Molecular Cardiovascular Physiology, Department of Biology, Ecology and Earth Sciences (Di.B.E.S.T.), University of Calabria, 87036 Rende, Italy; carmine.rocca@unical.it (C.R.); anna.de_bartolo@unical.it (A.D.B.); 3 College of Pharmaceutical Sciences, Soochow University, Suzhou 221400, China; 20197250001@stu.suda.edu.cn; 4 Department of Molecular Pharmacology, Albert Einstein College of Medicine, Forchheimer 209, 1300 Morris Park Avenue, Bronx, NY 10461, USA; michael.aschner@einsteinmed.org; 5 Department of Experimental and Clinical Medicine, Magna Graecia University of Catanzaro, 88100 Catanzaro, Italy; 6 National Institute of Cardiovascular Research I.N.R.C., 40126 Bologna, Italy; 7 State Key Laboratory of Quality Research in Chinese Medicine, Institute of Chinese Medical Sciences, University of Macau, Avenida da Universidade, Taipa, Macao 999078, China; annacheang@um.edu.mo

**Keywords:** apigenin, antioxidants, flavonoids, intracellular signalling, metabolic syndrome

## Abstract

Metabolic syndrome and its associated disorders such as obesity, insulin resistance, atherosclerosis and type 2 diabetes mellitus are globally prevalent. Different molecules showing therapeutic potential are currently available for the management of metabolic syndrome, although their efficacy has often been compromised by their poor bioavailability and side effects. Studies have been carried out on medicinal plant extracts for the treatment and prevention of metabolic syndrome. In this regard, isolated pure compounds have shown promising efficacy for the management of metabolic syndrome, both in preclinical and clinical settings. Apigenin, a natural bioactive flavonoid widely present in medicinal plants, functional foods, vegetables and fruits, exerts protective effects in models of neurological disorders and cardiovascular diseases and most of these effects are attributed to its antioxidant action. Various preclinical and clinical studies carried out so far show a protective effect of apigenin against metabolic syndrome. Herein, we provide a comprehensive review on both in vitro and in vivo evidence related to the promising antioxidant role of apigenin in cardioprotection, neuroprotection and renoprotection, and to its beneficial action in metabolic-syndrome-dependent organ dysfunction. We also provide evidence on the potential of apigenin in the prevention and/or treatment of metabolic syndrome, analysing the potential and limitation of its therapeutic use.

## 1. Introduction

Public-health-related chronic diseases, such as diabetes, cancer, depression, hypertension, stroke, Alzheimer’s disease and metabolic syndrome are prevalent worldwide. Both healthy diet and physical activities can combat or delay these diseases [1,2,3]. A major contribution to the healthy diet is provided by fruits and vegetables, which are rich in natural bioactive chemical compounds. Phytochemicals are a vital part of a healthy diet and have been shown to play a predominant role in combating these chronic diseases. There are numerous phytochemical compounds, such as polyphenols, alkaloids, tannins and glycosides, which have been described as having no toxicity but high therapeutic potential in several disease settings. Polyphenols are the largest contributing group within natural product compounds consisting of subgroups such as flavonoids, flavanones, isoflavonoids, flavones and flavanols [1,4,5,6,7].

Flavonoids are the secondary metabolites which have widespread metabolic functions in plants. They are widely distributed in fruits, flowers, seeds, vegetables and in both legumes and non-legume plants. More than 6000 flavonoid compounds have been isolated and investigated for different biological purposes and the number is constantly increasing. They are known to have diverse pharmacological activities, acting as anticancer, antioxidant and anti-inflammatory agents, and also inhibiting platelet aggregation and viral replication [6,7,8].

Moreover, several studies have indicated that the consumption of dietary flavonoids can be associated with a reduced risk of metabolic syndrome. Metabolic syndrome is considered to be a worldwide epidemic complex disorder that is defined by a cluster of interconnected factors that are able to significantly augment the risk of several cardiovascular diseases (CVD), including coronary heart disease (CHD), atherosclerosis and diabetes mellitus type 2 [9]. Thanks to their antioxidative and anti-inflammatory effects, flavonoids have been widely studied for the prevention and/or amelioration of metabolic syndrome and its related disorders. However, the beneficial effects of flavonoids need to be corroborated, also contextualizing their effects with the different individual risk factors associated with metabolic syndrome, as well as the different effects of flavonoids and their metabolites on the body [10].

Among the flavonoids, apigenin is one of the most investigated and isolated compounds in human health. It is abundant in plants such as parsley, onion, oranges, chamomile, celery, spices and honey and some plant-origin beverages such as wine, tea and beer. Diverse biological effects of apigenin have been reported in both in vitro and in vivo studies and several studies revealed that apigenin could display anti-inflammatory, antioxidant and anti-obesity actions, as well as antiproliferative and anticancer activities [11,12,13]. On the other hand, emerging evidence established a potential interaction between apigenin and human gut microbiota, which contains enzymes that could degrade apigenin; this is of particular interest in the context of metabolic disorders, since, as revealed by several studies conducted on humans and animal models, gut microbiota may exert a strong influence in the pathogenesis of metabolic syndrome. Accordingly, an imbalance between gut microbes and the host’s immune system could induce “metabolic endotoxemia”, leading to systemic inflammation and insulin resistance [14]. The intense investigation carried out over the last twenty years regarding the possibility of manipulating gut microbiota through dietary polyphenols for preventing metabolic disorders suggests that apigenin can also play a beneficial role. To date, although the potential activity of dietary apigenin for the modulation of the colonic microbial population composition or activity is still limited, some studies demonstrated that apigenin can affect both the growth and gene expression of selective gut bacteria strains, paving the way for further investigation [15,16].

Although extensive basic and clinical research is warranted to assess the relevance of apigenin in metabolic syndrome, an increasing number of studies provided evidence regarding the direct metabolic involvement of apigenin and its potential in ameliorating metabolic diseases, which is mainly attributed to its capacity to inhibit oxidative stress, regulate glucose and lipid metabolism and attenuate mitochondrial dysfunction. However, the metabolic implication of apigenin in pathophysiological conditions has not yet been fully elucidated. Therefore, the present review aims to summarize the findings, in particular obtained over the last five years, regarding the growing metabolic implications of apigenin and its beneficial effect against metabolic syndrome.

## 2. Chemistry and Biosynthesis of Apigenin

**Figure 1:**
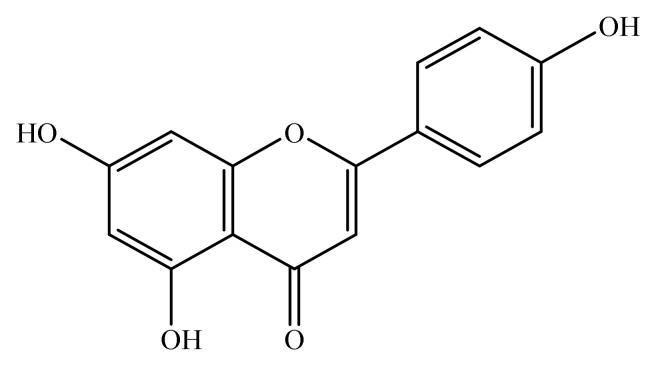
Chemical structure of apigenin (4′,5,7-trihydroxyflavone).

Apigenin is a natural bioactive flavonoid. Chemically, it is 4′,5,7-trihydroxyflavone (Figure 1) and is basically related to flavone, a subclass of flavonoids [17]. Apigenin is widely distributed in the plant kingdom, and is found in vegetables such as onions and parsley, fruits such as oranges and grape fruits and plant-derived beverages such as wine and tea. Apigenin is richly found in the plant species belonging to family Achillea, Sideritis, Fabaceae, Asteraceae, Teucrium, Genista, Artemisia, Matricaria and Lamiaceae [18,19,20,21,22].

**Figure 2:**
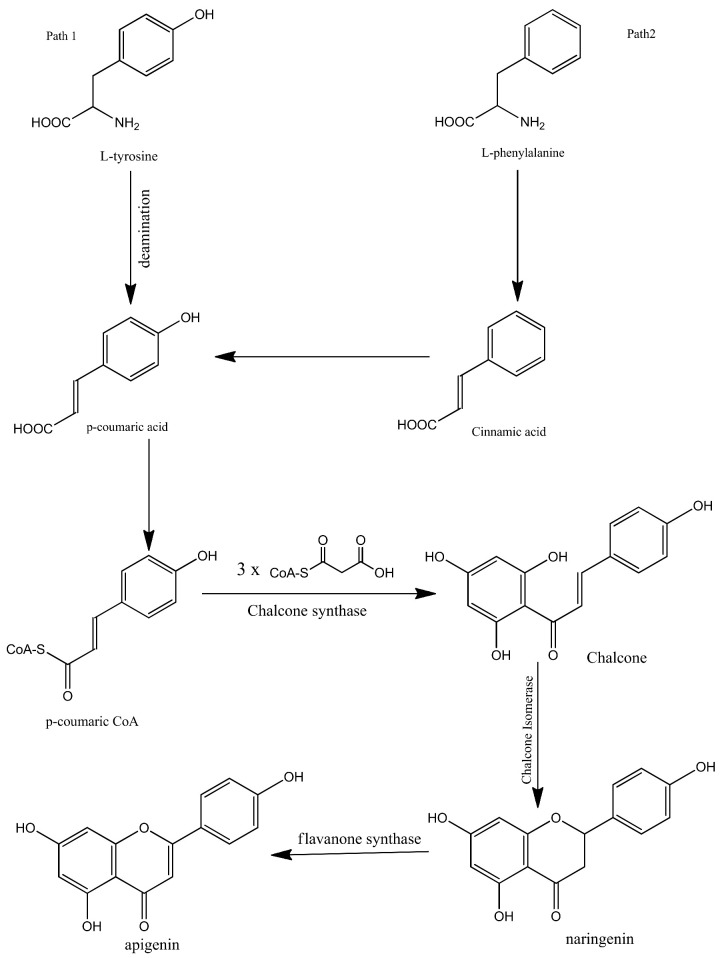
Biosynthesis of apigenin.

Apigenin is synthesized biogenetically by the phenylpropanoid pathway. It is synthesized from tyrosine and phenylalanine precursors. Tyrosine is directly deaminated to *p*-coumaric acid while phenylalanine is converted into cinnamic acid via non-oxidative deamination which is further oxidized at C-4 and then converted into *p*-coumaric acid. After the synthesis of *p*-coumaric acid by both pathways, the synthesized *p*-coumaric acid is further activated with Coenzyme-A. Three molecules of malonyl-CoA are then condensed with the *p*-coumarate. The compound is then aromatized to chalcone by chalcone synthase. Chalcone isomerase then isomerizes the chalcone and converts it into naringenin. In the final step, naringenin is oxidized into apigenin by flavanone synthase. The biosynthesis of apigenin is summarized in Figure 2 [1,23,24,25].

## 3. Pharmacokinetics of Apigenin

Apigenin is characterized by poor systemic availability, due to its low lipid and water solubility; it is either excreted unabsorbed in the urine or faeces or rapidly metabolized after absorption [16].

### 3.1. Absorption

Absorption of apigenin takes place throughout the gastrointestinal (GI) tract from stomach (deglycosylated) to colon (non-deglycosylated). Apigenin is transported via active carrier transport and passive diffusion in the duodenum and jejunum, while in the ileum and colon it is transported only by passive diffusion [26,27]. It has been established by Chen et al. that systemically absorbed apigenin was in conjugated form [28]. Li et al. found that topically administered apigenin was absorbed into local skin tissues despite transdermal blood circulation [29].

### 3.2. Distribution

The presence of apigenin was not found in the kidneys and GI lumen after 12 h of administration of apigenin glycosides (flavonoid extract). In the liver it was detected in 1.5 and 12 h [27]. Orally administered single-dose radiolabelled apigenin was detected after 10 days with radioactivity of 0.4% in kidneys, 1.2% in liver, 9.4% in intestine, 12% in faeces and 51% in urine [30]. Apigenin was also detected in human red blood cells [31].

Zhang et al. [32] (2013) indicated that apigenin binds to human serum transferrin glycoprotein at the Fe^3+^-binding site. Because of the high plasma binding capacity, apigenin distributes to tissues well and has a large volume of distribution in vivo [33].

### 3.3. Metabolism

Due to the poor bioavailability of apigenin, complete metabolism of apigenin occurs. The metabolism of apigenin occurs through phase II conjugation (sulfation and glucuronidation) reaction. Phase II biotransformation of apigenin involves both enteric and enterohepatic cycling [34]. Gradollato et al. found, in the rat liver, that the metabolism of apigenin follows phase I process via cytochrome P_450_ and nicotinamide adenine dinucleotide phosphate (NADPH). This phase I was followed by phase II process (glucuronidation and sulfation). One metabolite from sulfation and 3 β-monoglucuronides were seen after glucuronidation reaction [30]. The metabolites of both reactions were detected in plasma. Some other studies reported that sulfation of apigenin was less as compared to glucuronidation in both in vitro and in vivo assays [35].

Apigenin is metabolized by conjugation reactions. Prior to absorption of apigenin into blood and liver, the extensive conjugation of apigenin takes place in the gastrointestinal tract [36]. Some studies have reported that apigenin is also metabolized by hydrolysis in the liver and intestine. Deglycosylation of apigenin glucosides occurs in the epithelial β-glucosidases. Liver microsomes also metabolize apigenin [37].

### 3.4. Excretion

Apigenin is mostly excreted in urine and faeces. In particular, after a single oral dose of apigenin, 51.0% and 12.0% of apigenin were detected in urine and faeces. Most of the administered apigenin is excreted in unabsorbed form [30].

### 3.5. Bioavailability

Apigenin has been categorized as a class II drug with high intestinal membrane permeability and poor solubility according to the Biopharmaceutics Classification System [38]. Apigenin is poorly soluble in non-polar solvents (0.001–1.63 mg/mL), while it is soluble as more than 100 mg/mL (freely) in dimethylsulfoxide. The solubility in the phosphate buffer was 2.16 μg/mL at pH 7.5. Innovative techniques are used to enhance the bioavailability of apigenin [26,39]. For instance, carbon nanopowders with apigenin system was used and it was found that dissolution of apigenin was improved about 275% as compared to pure apigenin in 60 min, resulting in an increase of apigenin bioavailability of 183% [40]. In vitro assays reported that apigenin nanocrystals have faster dissolution velocity than coarse powder. These are prepared by a supercritical antisolvent process [41].

## 4. Metabolic Syndrome: An Overview

Metabolic syndrome is a combination of interlinked different clinical, physiological, biochemical and metabolic disorders that enhances the chances of atherosclerosis, type 2 diabetes mellitus and cardiovascular disorders, eventually leading to death [11]. Different factors such as high blood pressure, atherogenic dyslipidaemia, visceral adiposity, insulin resistance, genetic susceptibility and endothelial dysfunctions are involved in the pathogenesis of metabolic syndrome. These factors are interconnected by various mechanisms and pathways [42,43]. Metabolic syndrome has adverse effects on different body systems as summarized in Table 1.

According to the survey of International Diabetic Federation (IDF) and findings from the third National Health and Nutrition Examination Survey, 25% of the total adult population of the world succumbs from metabolic syndrome. According to the National Health and Nutrition Examination Survey, the prevalence of metabolic syndrome is found to be 60% in obese individuals, 22% in overweight and 5% among normal weight individuals [42,49,50,51]. However, it should be noted that the prevalence of metabolic syndrome varies across different racial/ethnic populations depending on the definitions used [9]. For instance, as systematically reviewed by Adjei et al., a higher prevalence of metabolic syndrome has been registered in Europe among migrants/ethnic minorities than host populations; in addition, a higher prevalence of metabolic syndrome has been reported in non-Hispanic Whites compared to Black populations. Furthermore, in the USA, some racial/ethnic differences have been found [52]. Therefore, a careful consideration of the influence of the race/ethnic group is needed in defining the prevalence and incidence of metabolic syndrome.

Metabolic syndrome is diagnosed by the definitions of World Health Organization (WHO), European Group for the Study of Insulin Resistance (EGIR), International Diabetes Federation (IDF) and American Heart Association/National Heart, Lung, and Blood Institute (AHA/NHLBI) as shown in Table 2. In addition, criteria for the metabolic syndrome diagnosis as represented by the National Cholesterol Education Program’s Adult Treatment Panel III report (ATP III) were evaluated in comparison to IDF. The latter was found to be more accurate [53].

In the context of the multiple derangements characterizing metabolic syndrome, insulin resistance, dyslipidaemia and hypertension represent important features that confer an increased risk for CVD and type 2 diabetes mellitus [55,56]. Individuals with insulin resistance show intolerance or impaired glucose metabolism characterized by excessive arterial blood pressure, increased fasting glucose level or a decrease in the actions of intravenous administered insulin, such as suppression of endogenous glucose production or insulin-mediated glucose clearance [57,58]. Dyslipidaemia is a spectrum of lipid abnormalities characterized by distortion in the structure, composition, biological activity and metabolism of atherogenic and anti-atherogenic lipoproteins and includes increased levels of triglycerides, elevated apolipoprotein B, decreased levels of high-density lipoprotein (HDL) cholesterol and elevated levels of small particles of low-density lipoproteins (LDL). Atherogenic dyslipidaemia is caused by insulin resistance via different mechanisms. Various metabolic abnormalities such as obesity, dyslipidaemia and glucose intolerance are commonly associated with essential hypertension [59]. Several studies have shown that the renin-angiotensin system is activated by hyperinsulinemia and hyperglycaemia via expressing angiotensin receptors, which lead to hypertension associated with insulin resistance [60].

It is noteworthy that human visceral obesity also represents a major risk factor for obesity-related diseases. Obesity is also responsible for the increased release of several biologically active substances, including adipokines, which act as classic hormones, thus exerting systemic metabolic effects; adipokines and cytokines may impair the insulin sensitivity of tissues and induce inflammation and chronic complications [61]. Therefore, a dysregulated signalling of adipokines may lead to metabolic disorders and chronic complications during obesity, representing a crucial mechanism by which dysfunctional adipose tissue promotes type 2 diabetes mellitus and CVD [61,62].

**Figure 3:**
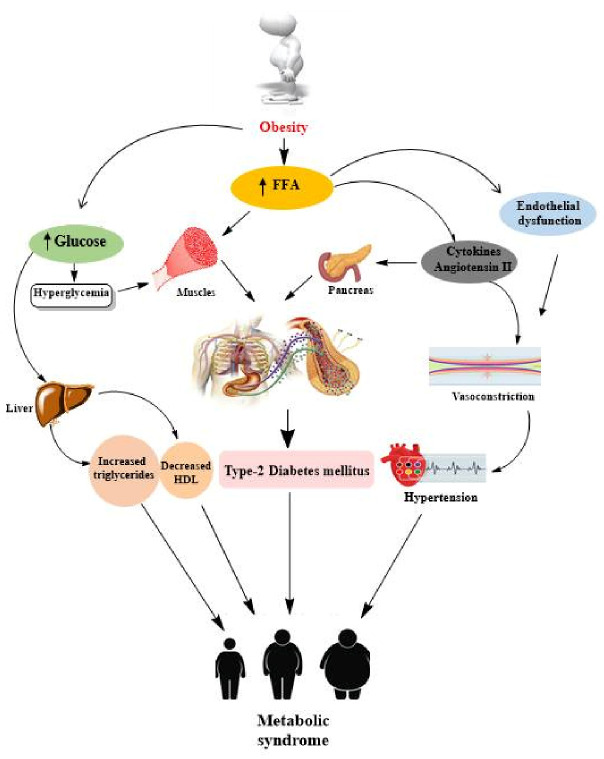
Pathophysiology of metabolic syndrome. Obesity, hypertension and insulin resistance are the major contributing factors in metabolic syndrome. FFA: free fatty acids; high-density lipoprotein (HDL) cholesterol.

A schematic representation of the pathophysiology of metabolic syndrome is depicted in Figure 3.

## 5. Broad-Spectrum Antioxidant Activity of Apigenin with a Main Focus on Dysmetabolic Conditions

It is widely accepted that flavonoids possess free-radical scavenger activity acting as potent antioxidant agents due to their polyphenolic structure [63]. Accordingly, epidemiological studies indicate that a diet rich in flavonoids and flavones has potential anticancer effects, especially for the digestive tract, prostate, pancreas, breast, haematological and skin cancer, and is associated with reduced risk of other chronic diseases, including CVD and neurodegenerative disorders [64,65,66]. Although in flavanones and flavones the hydroxyl group at position 3 and the catechol structure in the B-ring are absent, in reference to apigenin, the double bond at 2,3 carbon makes its structure more reactive, resulting in an antioxidant compound [67]. Moreover, apigenin scavenges superoxide, singlet oxygen and hydroxyl radicals in vitro [68]. Several research studies have proven the potential capacity of apigenin to act as a health-promoting agent [1 and references therein]. This natural flavone has been shown to exert therapeutic effects in neurological disorders and CVD [17]. Other studies showed that apigenin has hepatoprotective, antidepressant, anticancer and antihypertensive effects; it is also able to prevent autoimmune myocarditis, atherogenesis and asthma and induces beneficial action against osteoporosis, type 2 diabetes mellitus and pancreatitis [69,70,71,72,73,74,75,76,77,78,79,80]. Importantly, in addition to the involvement of apigenin in inflammation modulation, mRNA splicing regulation, cell cycle arrest and regulation of apoptosis [81], most of these findings support the hypothesis that the beneficial effects of apigenin are also attributed to its capacity to counteract oxidative stress, which is known to be a key component of many diseases, including cardiovascular, pulmonary, neurodegenerative diseases, cancer and metabolic disorders [82]. In particular, many studies strongly support the concept of the crucial impact of oxidative stress in the complex pathogenesis of metabolic syndrome and its major clinical manifestations (atherosclerosis, hypertension and diabetes), responsible for mitochondrial dysfunction, ectopic lipid accumulation and alteration of gut microbiota [83]. Therefore, a therapeutic approach targeting oxidative stress may ameliorate the risk factors associated with metabolic syndrome, delaying or preventing the progression of metabolic disease complications.

In the following sections, we provide information about the in vitro and in vivo antioxidant potential of apigenin in protecting different organs (i.e., heart, brain and kidney) against several toxic stimuli in order to introduce its beneficial action against some alterations in the above organs, which often occur during metabolic syndrome, before addressing the direct involvement of apigenin in dysmetabolism and metabolic syndrome.

### 5.1. Cardioprotective Potential of Apigenin through Antioxidant Action

Over the last five years, a large amount of in vitro and in vivo evidence indicates that apigenin has a broad-spectrum efficacy against oxidative-stress-dependent tissue/organ (including the heart) damage, since it has been shown to exert both direct antioxidant effect, by counteracting ROS formation, and indirect action by regulating the endogenous antioxidant defence system. It is known that oxidative stress is dramatically implicated in the onset and in the progression of CVD, such as cardiac arrhythmia, atherosclerosis, myocardial ischaemia/reperfusion (I/R) injury and heart failure (HF) [84,85]. Therefore, apigenin was extensively studied for its potential in attenuating cardiac injury from different types of insult by preserving heart function and regulating the oxidative cell balance. For instance, apigenin induces cardioprotection against myocardial hypertrophy by employing isoproterenol (ISO), widely used to induce cardiac hypertrophy and dysfunction in vitro and in vivo [86,87]. Buwa et al. (2016) demonstrated that apigenin can mitigate the hemodynamic, biochemical and histopathological changes in an ISO-induced rat model of myocardial infarction mainly by regulating the antioxidant defence system and improving the action of proliferator-activated receptor gamma (PPARγ) receptor signalling, which regulates the inflammatory response and may represent a potential therapeutic target to prevent cardiac hypertrophy and HF during metabolic disorders [88]. On the other hand, Thangaiyan and colleagues (2018) showed the beneficial action of apigenin on the single cardiomyocyte component since it prevented ISO-induced lipid peroxidative levels, DNA damage and antioxidant depletion in H9c2 cardiomyocytes [89]. These finding were corroborated by very recent data that evaluated the involvement of apigenin in the onset and progression of hypertension and hypertension-induced cardiac hypertrophy. In spontaneously hypertensive rats (SHRs), Gao et al. (2021) have found that bilateral hypothalamic paraventricular nucleus (PVN) chronic infusion of apigenin attenuated the increase of mean arterial pressure, cardiac hypertrophy and fibrosis, positively affecting PVN oxidative stress due to its ability to modulate the NADPH oxidase-dependent ROS generation and to restore the activity of superoxide dismutase 1 (Cu/Zn-SOD) and the 67-kDa isoform of glutamate decarboxylase (GAD67) [90].

It is widely known that anthracyclines (ANTs), including doxorubicin (DOX), are among the most prescribed chemotherapeutic drugs because of their strong efficacy in both solid and haematological tumours; however, their clinical use is hampered by dose-dependent and cumulative cardiotoxicity [91]. In the context of the complexity of ANTs-induced multifactorial cardiotoxicity, redox cycling and ROS generation play an undoubted role, although they do not represent the single and/or the principal event [92]. Interestingly, in addition to the recognized ability of apigenin in increasing DOX efficiency in different cancer cell lines, promising experimental data have recently been obtained on its cardioprotective role following DOX treatment that is mediated, at least in part, through reduction of oxidative stress. This is the case of the study carried out by Zare et al. (2019) [93] and Sahu et al. (2019) [94] and as reviewed by Navarro-Hortal et al. (2020) [95].

Apigenin has also been shown to precondition the heart against susceptibility to infarction; in particular, this flavone reversed vascular endothelial dysfunction in a cell culture model of early atherosclerosis, suppressing arterial ROS production and oxidative stress and protecting the heart against I/R damage [96]. In this regard, nutritional preconditioning with apigenin improved cardiac function in rats exposed to I/R by decreasing lipid peroxidation and enhancing SOD, glutathione peroxidase (GPX) and catalase (CAT) myocardial activities in a mechanism that targets a mitochondrial pathway mediated by the Notch1/Hes1 signalling [97]. Besides, apigenin reduced the oxidative stress and ROS production in I/R-induced H9c2 cells by activating the PI3K/Akt pathway [98].

#### Role of Apigenin in Dysmetabolism-Dependent Heart Dysfunction

The endothelial protective effect of apigenin was further evaluated in selective in vitro and in vivo models of endothelial dysfunction (ED), raising the hypothesis that this flavone could also be beneficial during dysmetabolic conditions. Indeed, ED is a key event in the progression of atherosclerosis, representing a hallmark of diabetes mellitus and obesity, which contributes to CVD [99]. ED in CVD is the major cause of the initial lesion in the vessel, representing an early damage marker for the development of vascular complications in diabetes; thus, the preservation of endothelial integrity is crucial for preventing diabetic complications. Regarding this aspect, interesting data have been obtained by Qin et al. (2016), who provided direct evidence that apigenin protected endothelial cells against high glucose-induced ED; mechanistically, apigenin improved the endothelium activity via inhibiting phosphorylation of protein kinase C βII (PKCβII) expression and downstream ROS production, and restoring the NO depletion via increasing eNOS activity [100]. Xu and collaborators (2021) provided evidence regarding the capacity of intragastric administration of apigenin to significantly ameliorate lipid profile by reducing total cholesterol, triglyceride and LDL cholesterol, and prevent atherosclerosis in hyperlipidaemic rats. Interestingly, apigenin also suppressed LDL oxidation and regulated the expression levels of lectin-like oxidized low-density lipoprotein (LDL) receptor-1 (LOX-1), Bcl-2 and Bax in the aorta of hyperlipidaemic rats, suggesting a promising anti-atherosclerotic role of apigenin in vivo [101].

Other studies moved towards a possible clinical application of apigenin for the treatment of myocardial complications during diabetes mellitus by employing in vivo models. Streptozotocin-treated rats exposed to myocardial infarction chronically treated with apigenin exhibited an improved haemodynamic function and a recovered redox status; by a pharmacological approach, the authors also found that apigenin acts through PPARγ stimulation [102]. As above reported, PPARγ may be considered a novel therapeutic target to prevent the cardiac complications and HF in metabolic disorders, since PPARγ agonists exhibited beneficial action against CVD [88]. Therefore, the ability of apigenin to significantly modulate PPARγ activity should be regarded with interest. Accordingly, other flavonoids modulate PPARγ signalling, thereby regulating the glucose/lipid metabolism [103] and corroborating the metabolic involvement of apigenin during CVD. It has been recently reported that hypoxia inducible factor (HIF)-lα represents a key regulator of glucolipid metabolism and is involved in the conversion of hypoxic myocardial energy utilization [104]. Notably, HIF-lα, strongly expressed in hypertrophied hearts, can be inhibited by apigenin; this finding, together with the ability of apigenin to selectively modulate PPARγ, corroborates the important role of apigenin in affecting blood pressure and glucolipid metabolism. In this regard, a recent study [105] examined the chronic effects of apigenin in a rat model of cardiac hypertrophy induced by renovascular hypertension, in which selective metabolic changes in myocardial energy utilization, based on the switch from fatty acids to glucose, occur [106]. The authors highlighted the beneficial contribution of apigenin in improving hypertensive cardiac hypertrophy and abnormal myocardial glucolipid metabolism by reducing myocardial HIF-1α expression and upregulating the expressions of myocardial PPARγ and its target genes, glycerol-3-phosphate acyltransferase genes (GPAT) and glucose transporter (GLUT-4) [105].

Other evidence emerges from the study of Liu et al. (2017), who demonstrated that apigenin administration improved the cardiac functions and mitigated the cardiac hypertrophy and interstitial fibrosis in a mouse model of diabetic cardiomyopathy by attenuating myocardial oxidative stress (i.e., 4-hydroxynonenal and malondialdehyde) and improving the activity of SOD and GPX [107]. The beneficial effects of apigenin on glucose and lipid metabolism are quite established, since apigenin improves glucose intolerance in miRNA103-overexpressing transgenic mice [108] and increases NAD^+^ levels through inhibition of cluster of differentiation 38 (CD38) in obese mice, ameliorating glucose and lipid homeostasis [109]. It is noteworthy that it has been shown that targeting NAD^+^ pathway may represent a strategy to ameliorate metabolic dysfunction since an increase in tissue intracellular NAD^+^ levels can protect against obesity, metabolic syndrome and type 2 diabetes [110,111]. However, further studies are necessary to better decipher and corroborate the cardioprotective role of apigenin by affecting metabolism during diabetic cardiomyopathy.

### 5.2. Neuroprotective Role of Apigenin through Antioxidant Action

In very recent years, apigenin showed great potential in counteracting neurodegenerative diseases and other brain disorders, offering neuroprotection through multiple mechanisms, including antioxidant action [112]. Focusing on the involvement of apigenin in redox regulation, the study by Shao and collaborators (2020) identified, by screening analyses, apigenin as a possible candidate to act as an antiepileptic agent [113]. By developing a two-photon fluorescence probe for the real-time tracking of endogenous hypochlorite fluxes in vivo and ex vivo in the brain of kainic-acid-induced epileptic mice, the authors demonstrated that apigenin was effective in reducing the expression of intracellular myeloperoxidase, a crucial source of free radicals generating oxidative stress in epileptic brains, and in regulating the activity of glutathione peroxidase 4 (GPX-4), thioredoxin reductase (TXNRD), glutathione (GSH) and SIRT1, thereby conferring neuroprotection through regulation of kainic-acid-induced ferroptosis and oxidative stress [113].

Diverse studies have indicated that ROS contributes to the disruption of the blood–brain barrier by disturbing tight junction proteins [114]; moreover, oxidative stress alters the amyloid β-peptide kinetics and is a major factor responsible for its toxicity [115]. In view of the powerful antioxidant effect of apigenin, in the last few years, several studies have shown its promising effect during neurodegeneration. Extensive literature data on both experimental and clinical models have been elegantly reviewed by Nabavi et al. (2018) concerning these aspects, pointing out apigenin as a promising bioactive compound for which future clinical trials should be encouraged to target neurodegenerative disorders [112].

Very recent studies [116,117,118] also explored the antioxidant protective role of apigenin in experimental models of ischemic stroke and cerebral I/R damage and subarachnoid haemorrhage. In both in vivo (murine models of middle cerebral artery occlusion) and in vitro (an oxygen-glucose deprivation/reoxygenation model of human brain microvascular endothelial cells and PC12 cells exposed to cobalt chloride) models, apigenin attenuated brain damage and neurological deficiency and improved neurological function, supporting previous evidence and indicating its capacity to ameliorate in vivo post-stroke cognitive impairments through regulating histone deacetylase and brain-derived neurotrophic factor (BDNF) [119].

#### Apigenin in Dysmetabolism-Dependent Brain Alteration

Starting from the assumption that apigenin is able to bind and interact with dipeptidyl peptidase-IV (DPP-IV), inhibiting its activity in the hippocampus of high fat, high fructose diet (HFFD)-fed insulin-resistant rats [120], Kalivarathan et al. (2017) demonstrated that apigenin exerted antioxidant action, improving antioxidant enzymes (i.e., SOD, CAT and GPX) and reducing the oxidative stress-related kinases (i.e., kappa B kinase beta (IKKβ) and c-Jun NH2 terminal kinase (JNK), and the nuclear translocation and activation of nuclear factor kappa B (NF-κB)) activation in the hippocampus of HFFD-fed rats [120]. These finding are in accordance with another recent report that extended the knowledge of the molecular mechanism by which apigenin protects the brain during dysmetabolism; the authors found that similarly to sitagliptin (STG), a selective DPP-IV inhibitor, apigenin can improve cognitive function by activating cAMP response element-binding protein (CREB)-BDNF axis and by improving brain insulin signalling in HFFD-fed rats [120]. Notably, STG is approved for the treatment of type 2 diabetes mellitus due to its effect in improving glucose and lipid metabolism and insulin sensitivity; STG is also known to exert neuroprotective action in insulin-resistant rats due to its ability to ameliorate recognition memory, oxidative stress and hippocampal neurogenesis [121]. Considering the ability of apigenin to act as a DPP-IV inhibitor with an efficiency comparable to STG, it is conceivable to hypothesize that apigenin could be useful in counteracting neurodegenerative events associated with insulin resistance and metabolic syndrome. Of course, additional studies need to be performed in order to establish the involvement of apigenin in dysmetabolic-dependent neurological impairment.

### 5.3. Renoprotection of Apigenin against Oxidative Stress-Induced Injury

Thanks to its antioxidant action, apigenin can inhibit oxidative stress to attenuate the nephrotoxicity secondary to different toxic stimuli, including pharmacological agents such as cisplatin and DOX. Cisplatin is a highly effective antineoplastic drug that in combination chemotherapy regimens is currently used as front-line therapy in the treatment of several cancers; however, nephrotoxicity is a serious and dose-limiting toxicity of cisplatin [122]. Promising preventive effects of apigenin against cisplatin-induced nephrotoxicity have been obtained in experimental studies by different researches; in particular, Hassan et al. (2017) and Wu et al. (2021) showed that chronic administration of apigenin relieved the oxidative injury dependent on cisplatin due to its antioxidant effect and its ability to enhance SOD and GPX activities [123,124]. On the other hand, He et al. (2016) confirmed the protective effect of apigenin in a mouse model of DOX-induced nephrotoxicity [125]. Further studies are however necessary to evaluate whether apigenin could hamper the anticancer efficacy of these chemotherapeutics in vivo. The antioxidant capacity of apigenin in other kidney-related pathological contexts was confirmed by various recent studies. In this regard, Liu et al. (2017) showed that a 24 h pre-treatment with apigenin protected against 45 min of renal ischaemia, followed by 24 h of reperfusion in a rat model [126], while Wang and colleagues [127] have found that apigenin improved SOD, GSH and CAT enzyme levels to mitigate renal pathological changes in mice exposed to mesoporous silica nanoparticles, a promising drug delivery system whose toxicity has been documented [128]. Similar findings have been obtained by Ali et al. (2021), who showed the beneficial action of apigenin against kidney damage induced by nickel oxide nanoparticles (NiONPs) exposure in rats. NiONPs have important medical applications; unfortunately, they showed a significant toxic profile mainly due to pro-oxidative action [129]. Moreover, Zamani and collaborators indicated, in rats, that apigenin was effective in exerting renal mitochondrial protection and antioxidant action following the exposure of carbon nanotubes, which induce mitochondrial dysfunction and oxidative stress and accumulate in several organs and tissues, including kidneys, with consequent organ damage [130].

#### Apigenin in Renal Complication following Dysmetabolism

As above mentioned, and as will be more detailed in the next part of the present review, the antidiabetic activity of apigenin is extensively studied because of its capacity to regulate glucose metabolism, inhibit α-glucosidase activity and increase insulin secretion [1,131]. Conversely, the effectiveness of apigenin in ameliorating the renal complication following dysmetabolism was less addressed. One of the first studies that evaluated the action of apigenin in diabetic nephropathy was carried out by Malik and colleagues (2017) [132]. It is well known that diabetic nephropathy is one of the major microvascular complications of diabetes mellitus, where oxidative stress plays a predominant role in its progression [133]. In a rat model of streptozotocin-induced diabetic nephropathy, the authors indicate that apigenin significantly attenuated renal impairment by suppressing oxidative stress (as evinced by reduced MDA and increased SOD, CAT and GSH) and inhibiting MAPK pathway. The use of streptozotocin is widely reported for studying antioxidant potential of pharmacological and bioactive substances in hyperglycaemic contexts, since this drug selectively induces an excess of ROS formation via exertion of heavy electron pressure on the mitochondrial electron transport chain from oxidation of overproduced NADH [134]. Notably, the authors also found that the effects of apigenin were similar to those of ramipril, which induces renoprotection by blocking angiotensin type 1 receptor (AT1R)-mediated oxidative stress, indicating that apigenin is strictly involved in reducing free radicals’ formation and oxidative stress during diabetic nephropathy. Additional mechanistic knowledge regarding the renoprotection of apigenin in diabetic conditions was provided by a very recent study [135]. Here, the authors demonstrated that apigenin administration in Zucker diabetic fatty (*fa/fa*) rats effectively downregulated CD38 expression and mitochondrial oxidative stress, thus reducing tubulointerstitial fibrosis and tubular cell damage; this flavone also increased the kidney intracellular NAD^+^/NADH ratio and Sirt3-mediated mitochondrial antioxidant enzymes. Based on these findings, it is conceivable that apigenin plays a crucial role in the pathogenesis of diabetic kidney disease and that additional studies are warranted to elucidate its mechanism of action.

Table 3 recapitulates the main cardioprotective, neuroprotective and renoprotective effects of apigenin following different toxic stimuli and during dysmetabolic-induced organ dysfunction.

## 6. Effects of Apigenin in Metabolic Syndrome

As above mentioned, metabolic syndrome is a progression of health-related abnormalities which is characterized by dyslipidaemia, hypertension, insulin resistance and fatty liver. Metabolic syndrome worsens the pathological conditions of CVD and diabetes. [136,137,138,139,140]. An increasing number of studies confirm that oxidative stress is one of the leading causes of metabolic syndrome and ensuing pathological conditions such as diabetes and hypertension [138,141]. Therefore, treating or preventing oxidative stress can represent a promising strategy to attenuate insulin resistance and metabolic syndrome and the associated CVD and type 2 diabetes mellitus [1,142,143,144].

Several studies report that apigenin can be effective in the management of metabolic syndrome due to its ability to improve glucose and lipid metabolism during dysmetabolic conditions [1,131,145]. Nuclear factor erythroid 2-related factor 2 (Nrf2) is considered to be a key endogenous antioxidant transcription factor. Antioxidant proteins are expressed by the interaction of Nrf2 and antioxidant response element (ARE) in DNA [146,147]. Under normal conditions, Nrf2 forms a complex with the cytoplasmic Kelch-like ECH-associated protein 1 (Keap1) which results in proteolysis of Nrf2. When the Nrf2-Keap1 complex is exposed to an oxidant or electrophile, Nrf2 translocates into the nucleus and forms a complex with ARE. The Nrf2-ARE complex starts the transcription of numerous antioxidant genes. Dissociation of the Nrf2-Keap1 complex results in the activation of Nrf2, which further facilitates the nuclear transcription of antioxidant genes. Molecular docking coupled with expression studies have shown that apigenin interferes with the binding of Nrf2 and Kaep1 and thus dissociates Nrf2, increasing the latter’s nuclear translocation and antioxidant activity [138,148,149].

Emerging reports indicate that mammalian cells have key NAD^+^ ase in the form of CD38 and that the manipulation of NAD^+^ metabolism may represent a plausible strategy to ameliorate obesity, metabolic syndrome and type 2 diabetes. It has been reported that overexpression of CD38 leads to acetylation of proteins which results in the protein degeneration in obesity. Studies have shown that CD38 is abundantly found in mitochondria where the sirtuin-1 (SIRT1) activity and NAD^+^ ase level are maintained. In this regard, Escande et al. carried out in vivo analysis and confirmed the mechanistic pathways of apigenin as a remedy for the management of metabolic syndrome [109]. The inhibition of CD38 by apigenin can enhance the homeostasis of glucose and thus improve liver oxidation of fatty acids, with the observation that the apigenin-treated mice for 4 days had markedly decreased levels of blood glucose relative to the control mice [109]. The authors showed that CD38 is a crucial target site for controlling metabolic syndrome and found that apigenin acts as a CD38 inhibitor. Apigenin was administered to obese mice, raising the NAD^+^ levels in the liver by inhibiting CD38 and reducing protein acetylation. Lipid metabolism, glucose homeostasis and glucose tolerance were improved in obese mice by inhibiting CD38 [109,110,150,151]. Thus, apigenin appears to be a CD38 inhibitor that may be used as a promising strategy for the treatment of metabolic syndrome via activation of NAD+ and SIRT1.

Besides, Jung et al. evaluated the long-term supplementary effects of low-dose apigenin on obesity, establishing that this flavone is effective in treating metabolic syndrome in obese mice (induced by high fat diet) by ameliorating the insulin resistance, dyslipidaemia and hepatic steatosis [140]. They reported that apigenin significantly normalized the fasting blood glucose level by normalizing plasma insulin levels and the β-cell function. Apigenin also attenuated inflammation by significantly lowering the plasma concentrations of pro-inflammatory mediators like IFN-γ, IL-6, TNF-α and MCP-1. They also found that in apigenin-treated obese mice, the concentrations of plasma-free fatty acid were significantly reduced. Apigenin also markedly reduced the total plasma cholesterol levels, plasma apoB/A1 ratios and plasma apoB. Importantly, apigenin significantly decreased the activity of glucose-6 phosphatase and phosphoenolpyruvate carboxykinase (PEPCK), two key regulatory enzymes of gluconeogenesis [140,152]. It has been reported that an impairment of gluconeogenesis promotes hepatic glucose production, aggravating insulin resistance and further contributing to the pathogenesis of hyperglycaemia and glucose intolerance [152,153]. These data reinforce the hypothesis according to which apigenin improves insulin sensitivity and glucose tolerance, modulating hepatic gluconeogenesis. These findings are supported by the study of Cazarolli and collaborators who tested the effect of apigenin-6-C-(2″-O-α-L-rhamnopyranosyl)- β-L-fucopyranoside, obtained from Averrhoa carambola L. leaves, on ^14^C-glucose uptake. The authors showed that this compound was effective in lowering blood glucose and stimulated glucose-induced insulin secretion after oral treatment in hyperglycaemic rats acting as an insulin-mimetic agent [154].

Another important finding deriving from this and other studies regards the pancreatic beneficial action of apigenin during dysmetabolism. Pancreatic β-cells are very sensitive to oxidative stress, which is one of the major causes of cell damages in diabetes. Accordingly, in vivo and in vitro findings showed that pre-treatment with apigenin protected RINm5F pancreatic beta cells against streptozotocin-induced oxidative damages and decreased the intracellular ROS production, oxidative stress (i.e., lipid peroxidation and protein carbonylation) and cellular DNA damage and mitigated apoptosis of β-cells induced by streptozotocin [155].

### 6.1. Peroxisome Proliferator-Activated Receptor γ (PPARγ) as a Main Metabolic Target of Apigenin

Feng et al. performed two separate studies involving both in vivo and in vitro examinations which provided molecular insights into the apigenin-induced metabolic effect via targeting peroxisomes PPARγ [156,157]. In the first study, the authors showed that apigenin mitigated HFD-induced non-alcoholic fatty liver disease (NAFLD) progression, attenuating lipid accumulation and oxidative stress in mice; these effects were mainly attributed to the ability of apigenin to activate Nrf2 by promoting its translocation into the nucleus that, in turn, inhibited the function of PPARγ [156]. These findings are of significant interest since PPARγ, as a ligand-dependent transcription factor, is crucially involved in lipid metabolism and oxidative stress. Indeed, its results increased in fatty liver disease associated with obesity in both animal and human models and the inhibition/knockdown of PPARγ ameliorates fatty liver in obese and NAFLD conditions [158,159]. In the other study, the authors observed that apigenin ameliorated the insulin resistance, metabolic abnormalities and obesity-related inflammation by also acting on PPARγ [157]. It has been reported that PPARγ has a key role in the low-grade inflammation and adipogenesis in metabolic syndrome. PPARγ is a member of ligand inducible transcription factors (PPAR) and regulates the functional polarization of macrophages acting as anti-inflammatory regulators in atherosclerosis [160,161]. Macrophages can be found in a polarized inflammatory state (macrophages 1; M1) which generates inflammatory cytokines, such as interleukin-6 (IL-6), tumour necrosis factor (TNF) and IL-1R and effector molecules such as nitric oxide and ROS. Macrophages may also be found (macrophages 2; M2) in a polarized anti-inflammatory state (IL-10, IL-12 and IL-23). M1 macrophages have inherent activation of genes such as C-C chemokine receptor type 7 (CCR7) and CD38, while in M2, genes such as mannose receptors type C, arginase, chitinase-like Ym-1 and certain scavengers are activated [162,163]. Feng et al. established a relationship between macrophage polarization and apigenin and found that apigenin binds to PPARγ and alters the equilibrium between M1 and M2 polarization, thus inducing its biological actions in metabolic syndrome. In their in vitro cell model, they found that apigenin decreased the levels of CD80, CCR7, IL-6, TNF and nitric oxide in M1 cell model, while in M2 cell model, apigenin increased the levels of mRNA gene expression, IL-10 and arginase1 activity on the surface of macrophages. This in vitro analysis confirmed that apigenin favours the upregulation of M2 markers and downregulation of M1 markers. Thus, apigenin could inhibit the inflammatory macrophages (enhance polarization of M2), attenuating obesity-related inflammation [157]. Similarly, another in vitro study confirmed that apigenin binds to PPARγ, acting as allosteric effector. Salam et al. also confirmed that apigenin is a PPARγ agonist [164,165].

It should be noted that during adipocytes differentiation, PPARγ, in addition to CCAAT/enhancer binding protein-α (C/EBPα), represents a key transcription factor; PPARγ and C/EBPα act in concert to activate the gene expression of fatty-acid-binding protein and PEPCK and to promote lipid synthesis [166]. In this regard, the 3T3-L1 preadipocytes cell line is widely employed to reproduce an adipocyte differentiation model system for studying the metabolic mechanisms of obesity and the molecular mechanisms of adipogenesis since these cells differentiate into mature adipocytes in vitro [167]. It is noteworthy that in recent years, apigenin was also found to inhibit the differentiation of 3T3-L1 preadipocytes; in particular, apigenin exhibited marked synergistic effect together with emodin in both differentiation and pancreas lipase assays [168]. According to these reports, apigenin alleviated the glucose metabolic disorders in HFD mice [140] and abrogated weight gain potentiation after HFD re-exposure by improving gut microbes; interestingly, apigenin also increased energy expenditure with an increase in Ucp1 expression, suggestive of adipose tissue browning and increased non-shivering thermogenesis [169]. These data further corroborate the role of apigenin in obesity and support the conclusions of Escande et al. (2013), according to which apigenin regulates NAD^+^ levels and global protein acetylation through sirtuin-dependent metabolic disorders [109].

### 6.2. Additional Insight in the Mechanism Underlying Apigenin-Induced Metabolic Effects

Another research paper published in 2021 analysed the function and mechanism of apigenin during insulin resistance by reproducing in vitro and in vivo models (intracellular fat accumulation model cells using palmitate and HFD–fed mice); the authors observed that apigenin downregulated sterol regulatory element-binding protein 1c (SREBP-1c), sterol regulatory element-binding protein 2 (SREBP-2), fatty acid synthase, stearyl-CoA desaturase 1 and 3-hydroxy-3-methyl-glutaryl-CoA reductase and endoplasmic reticulum stress in vitro and in vivo; these effects were functionally associated with an improvement of lipid profile and insulin resistance [170]. The authors highlight a novel mechanism by which apigenin can mitigate hyperlipidaemia-related states.

Excessive accumulation of visceral fat inside the abdominal cavity (surrounding vital organs) is referred to as visceral obesity. Most evidence indicates that visceral obesity is directly related to metabolic syndrome. High mortality rate and greater risk of metabolic syndrome (insulin resistance, glucose intolerance, dyslipidaemia and hypertension) are associated with excessive visceral fat accumulation [171,172]. It has been observed that in the pathogenesis of visceral obesity, signal transducer and activator of transcription 3 (STAT3) is a key factor. STAT3 is constitutively active in obese individuals and stimulates the visceral adipose tissues [173]. Moreover, growing evidence indicates that increased levels of cluster determinant 36 (CD36) are directly linked to obesity. Deficiency of CD36 reduces the visceral fat accumulation and thus decreases the progression of obesity [174]. Interestingly, Tao et al. provide additional knowledge about the mechanism of action underlying the antivisceral obesity effect of apigenin; here, the authors found that apigenin can counteract metabolic syndrome by decreasing the visceral obesity via inhibiting CD36 and STAT3 signals and consequently reducing PPARγ expression in adipocytes [175]. These data indicate the STAT3/CD36/PPARγ-signalling axis as an important therapeutic target in the apigenin-mediated antivisceral and anti-obesity action.

**Figure 4:**
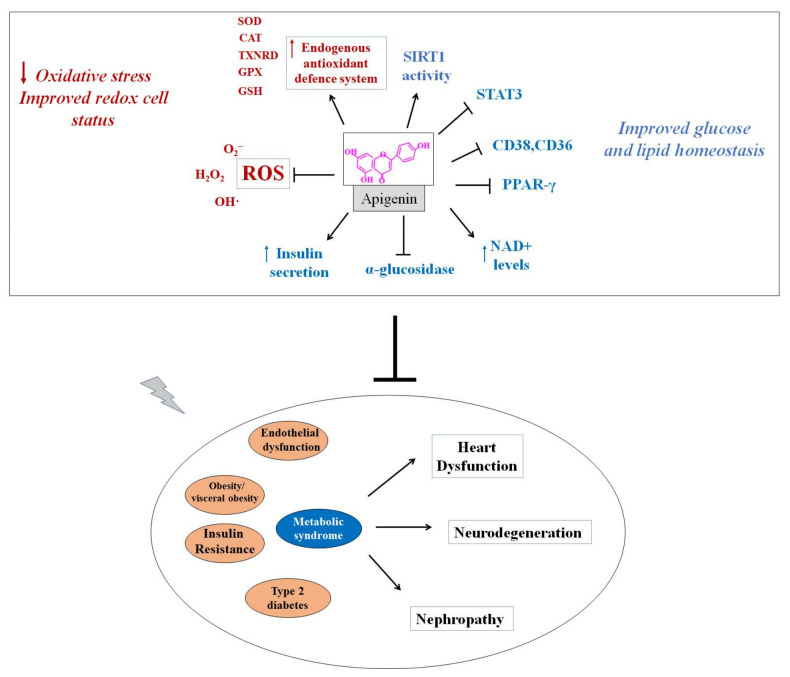
Proposed mechanism of action of apigenin in metabolic syndrome. CAT: catalase; CD36: cluster determinant 36; CD38: cluster of differentiation 38; GPX: glutathione peroxidase; GSH: glutathione; H_2_O_2_: hydrogen peroxide; O_2_^−^: superoxide anion; OH·: hydroxyl radical; SIRT1: sirtuin-1; SOD: superoxide dismutase; STAT3: signal transducer and activator of transcription 3; TXNRD: thioredoxin reductase.

The schematization depicted in Figure 4 recapitulates the antioxidant action and metabolic effects by which apigenin could improve glucose and lipid metabolism, ameliorating endothelial dysfunction and insulin resistance and reducing visceral obesity and hyperglycaemia, thus resulting in cardio-, neuro- and renoprotection during metabolic syndrome.

## 7. Does Apigenin Have Potential Clinical Impact in Metabolic Syndrome?

The potential clinical uses of apigenin have been evaluated for different diseases such as depression, insomnia, Alzheimer’s disease, cancer and amnesia. Of course, additional experimental and clinical studies are warranted to establish the real therapeutic impact of apigenin in the near future [1,112] and references therein. However, to date, most of the findings regarding the ability of apigenin in preventing and/or treating metabolic syndrome and its related complications are limited to pre-clinical models. Indeed, there is a lack of research related to the potential of apigenin in dysmetabolic conditions for humans. On the other hand, it should be noted that important targets taking part in the mechanism of action by which apigenin induces beneficial effect in dysmetabolism have been studied in clinical practice for the management of metabolic syndrome. For instance, Feng et al. have found that the homeostasis of macrophages 1 (M1) and macrophages 2 (M2) phenotypes is necessary for the management of inflammatory disorders. PPARγ inhibits the inflammation via activation of M2 phenotypes. PPARγ ligands have been established as stimulating the polarization of macrophages in anti-inflammation and insulin resistance. In clinical practice, PPARγ agonists such as pioglitazone and rosiglitazone have been used for a long time for the management of insulin resistance, which decreases the activation of MCP1, IL-6 and TNF and regulates the synthesis of adiponectin (anti-inflammatory molecule) [176,177]. Recently, clinical trials have been performed on PPARγ agonists such as rosiglitazone, pioglitazone (thiazolidinedione) and sitagliptin and metformin combination with rosiglitazone for the management of insulin resistance in metabolic syndrome. Though PPARγ agonists (thiazolidinedione) are approved for clinical practice, their use has been limited due to severe liver toxicity, bone disorders and cardiovascular side effects [178]. Therefore, researchers are now trying to find out natural compounds for the management of abnormalities associated with metabolic syndrome with high efficacy and lessened side effects. As it has been established earlier that PPARγ ligands have a promising role in the treatment of metabolic syndrome, therefore much more attention has been paid towards newer and safer natural PPARγ agonist compounds.

Apigenin has been established as a PPARγ ligand; moreover, Escande et al. have shown that apigenin is a NAD+ ase CD38 inhibitor, and Nicholas et al. found apigenin to be a modulator of NF-kB in macrophages which improves metabolic syndrome [109,157,165,179]. Feng et al. have shown that apigenin is effective in the management of metabolic syndrome via PPARγ with lesser side effects than thiazolidinedione. Overall, these results on pre-clinical models indicate that the effects of apigenin in metabolic syndrome are worthy of emphasis and investigation in future clinical studies.

## 8. Future Perspectives and Limitations

Bioactive compounds deriving from food and plants are used as supplements or nutraceutical products [180,181], but the challenging aspect of isolation of bioactive compounds, their bioavailability and stability, should be resolved via different techniques by recovering them from some other natural sources like microalgae. This challenging process has been overcome for apigenin by recovering it from agrofood wastes and microalgae using different techniques, such as microwave-assisted extraction, supercritical CO_2_ and subcritical water technologies and enzymatic techniques [182,183,184]. Researchers have found apigenin to be the safest therapeutic moiety. However, it has been reported that high doses (30 mg/kg, 100 mg/kg, 200 mg/kg) of apigenin may induce hepatocyte degeneration and toxicity, mild sedative effect and muscle relaxation in murine models [185,186,187]. Apigenin, being a potential therapeutic agent, needs to be evaluated in novel therapeutic formulations through nanodelivery and microdelivery techniques so as to enhance its therapeutic efficacy and target specificity [188,189].

## 9. Conclusions

Extensive research has provided indication of the impact of diet and lifestyle in preventing the onset of diseases that are strictly linked to negative lifestyle and dietary habits, such as metabolic syndrome. Therefore, lifestyle modifications can represent the main therapeutic strategy for the treatment and management of metabolic syndrome. Randomized controlled trial studies clearly indicated that selective dietary patterns based on Mediterranean dietary patterns (MDP), dietary approaches to stop hypertension (DASH) diet and Nordic diet exhibit beneficial action against metabolic syndrome. Their effect may be attributed to the high presence of fruits, vegetables, whole grains, fish and nuts [190]. Additional evidence reported that plant-based diets, if conducted according to dietary guidelines and recommendations, may significantly reduce the risk of coronary heart disease and other cardio-cerebrovascular diseases, as well as of metabolic syndrome and type 2 diabetes, indicating that these regimens can be effective in preventing and treating cardio-metabolic and metabolic diseases.

Many of these chronic diseases require pharmacological intervention, while others can be prevented by including selective nutraceuticals in the diet. Apigenin possesses diverse organoleptic and nutritional properties in natural phenolic compounds. Due to its countless therapeutic uses and beneficial health-related properties, it is important to further delineate the mechanism of actions of apigenin for its inclusion in nutraceutical formulations [12,191,192]. Experimental evidence from in vivo and in vitro reports and clinical studies showed that apigenin can be considered a promising therapeutic agent for the prevention and/or management of metabolic syndrome and its related risk factors. Although randomized controlled clinical trials indicate that apigenin can exhibit interesting neuroprotective properties against different neurological disorders (i.e., depression, Alzheimer’s disease and Parkinson’s disease) [1,112], there is a lack of research related to the potential of apigenin in dysmetabolic conditions and metabolic syndrome for humans. Therefore, additional cellular, animal models and clinical studies are mandatory to characterize the safety and efficacy of apigenin in reducing and/or slowing the onset of metabolic syndrome, mainly as a preventive agent able to act “beyond the diet but before the need to use a drug”.

## Figures and Tables

**Table 1:** Adverse effects of metabolic syndrome on different body systems.

Target Sites	Systemic Effects of Metabolic Syndrome	References
Skin	Psoriasis, systemic lupus erythematosus, alopecia, acne inversa, burn-induced insulin resistance, skin cancer	[44]
Eye	Open angle glaucoma, age-related cataract, oculomotor nerve palsy, central retinal artery occlusion, non-diabetic retinopathy	[42]
Liver	Hepatic fibrosis, non-alcoholic steatohepatitis, elevated serum transaminase, cirrhosis and non-alcoholic fatty liver	[45]
Kidney	Glomerulomegaly, microalbuminuria, focal segmental glomerulosclerosis, hyperfiltration, hypofiltration and chronic kidney disorder	[46]
Cardiovascular System	Myocardial infarction, stroke and coronary heart disease	[47]
Reproductive System	Erectile dysfunction, polycystic ovarian syndrome and hypogonadism	[48]

**Table 2:** Definitions and criteria for metabolic syndromes according to World Health Organization (WHO), European Group for the Study of Insulin Resistance (EGIR), International Diabetes Federation (IDF) and American Heart Association/National Heart, Lung, and Blood Institute (AHA/NHLBI). According to IDF, 25% of the total adult population of the world succumbs from metabolic syndrome. According to the National Health and Nutrition Examination Survey, the prevalence of metabolic syndrome is found to be 60% in obese individuals, 22% in overweight and 5% among normal weight individuals. * According to Alberti et al. (2009) [54], it is recommended that the IDF cut-off points be used for non-Europeans and either the IDF or AHA/NHLBI cut-off points be used for people of European origin until more data are available. HDL: high density lipoprotein, Rx: pharmacologic treatment, TG: triglycerides, BMI: body mass index; waist circumference in EGIR is for European men and women.

Criteria	WHO (1998)	IDF (2005)	EGIR (1999)	AHA/NHLBI (2009)
	Diabetes or Insulin Resistance + Two of the Four Criteria Below	Obesity + Two of the Four Criteria Below	Hyperinsulinemia + Two of the Four Criteria Below	3 of the Following 5 Risk Factors
**Hyperglycaemia**	Insulin resistance already required	Fasting glucose ≥ 100 mg/dL	Insulin resistance already required	Fasting glucose ≥ 100 mg/dL
**Hypertension**	≥140/90 mmHg	>130 mmHg systolic or >85 mmHg diastolic	≥140/90 mmHg	≥130 mmHg systolic or ≥85 mmHg diastolic
**Obesity**	Waist/hip ratio: >0.90 (M) > 0.85 (F) or BMI > 30 kg/m^2^	Central obesity already required	Waist circumference: ≥94 cm (M) ≥80 cm (F)	* Waist circumference: ≥102 cm (M) ≥88 cm (F) TG ≥ 150 mg/dL
**Dyslipidaemia**	TG ≥ 150 mg/dL or HDL-C < 35 mg/dL (M) and <39 mg/dL (F)	TG ≥ 150 mg/dL or Rx	TG ≥ 177 mg/dL or HDL-C < 39 mg/dL	HDL-C < 40 mg/dL (M) and <50 mg/dL (F)

**Table 3:** Cardioprotective, neuroprotective and renoprotective effects of apigenin following different toxic stimuli and during dysmetabolic-induced organ dysfunction.

Organ	Effects of Apigenin through Antioxidant Action	Effects of Apigenin during Dysmetabolic-Dependent Organ Alteration
	- Protection against ISO-induced myocardial hypertrophy and myocardial infarction in vitro and in vivo models [86,87,88,89]	- Protection against high glucose-dependent endothelial dysfunction in vitro [100]
	- Improvement of hypertension, cardiac hypertrophy and fibrosis in vivo [90]	- Amelioration of lipid profile and LDL oxidation in hyperlipidaemic rats [101] - Protection against diabetes-mellitus-induced cardiac alteration in vivo [102,103]
**HEART**	- Protection against DOX-dependent cardiotoxicity [91,93,94,95]	- Protection against hypertensive cardiac hypertrophy and abnormal myocardial glucolipid metabolism in vivo [105,106]
	- Improvement of vascular endothelial and cardiac function in vivo during aging or following I/R insult [96,97]	- Improvement of cardiac functions, cardiac hypertrophy and interstitial fibrosis in a mouse model of diabetic cardiomyopathy [107]
	- Protection against I/R-induced damage in H9c2 cells [98]	- Improvement of glucose intolerance in miRNA103-overexpressing transgenic mice and glucose/lipid homeostasis [108,109,110,111]
	- Neuroprotection against kainic-acid-induced ferroptosis and oxidative stress in vivo [113]	
**BRAIN**	- Improvement of brain damage and neurological deficiencies following ischemic stroke, I/R damage or subarachnoid haemorrhage [116,118,119]	- Protective hippocampal action in rats fed high fat, fructose diet [120]
**KIDNEY**	- Protection against cisplatin-induced nephrotoxicity or DOX-induced nephrotoxicity in vivo [123,124,125] - Beneficial preconditioning effect against renal I/R injury [126] - Protection against renal pathological changes in mesoporous silica-nanoparticles-treated mice [127] - Reduction of NiONPs-induced kidney damage and carbon-nanotubes-induced mitochondrial dysfunction in rat models [29]	- Protection against diabetic nephropathy in a rat model [132] - Improvement of tubulointerstitial fibrosis and tubular cell damage in Zucker diabetic fatty (*fa/fa*) rats [135]

ISO = isoproterenol, DOX = doxorubicin, LDL = low density lipoproteins, I/R = ischaemia/reperfusion, NiONPs = nickel oxide nanoparticles.

## Abbreviations

ARE	Antioxidant response element
AT II	Angiotensin II
ATP III	National Cholesterol Education Program’s Adult Treatment Panel III report
BMI	Body mass index
CAT	Catalase
CD36	Cluster determinant 36
CD38	Cluster of differentiation 38
CETP	Cholesterol ester transport protein
CHD	Coronary heart disease
C_max_	Maximum concentration
CVD	Cardiovascular diseases
EGIR	European Group for the Study of Insulin Resistance
FFA	Free fatty acids
GPX	Glutathione peroxidase
GSH	Glutathione
HDL	High-density lipoproteins
HF	Heart failure
I/R	Ischaemia/reperfusion
IDF	International Diabetes Federation
IL	Interleukin
IL-6	Interleukin 6
ISO	Isoproterenol
Keap1	Kelch-like ECH-associated protein 1
LDL	Low-density lipoproteins
M1	Macrophages 1
M2	Macrophages 2
MRP	Multidrug resistance proteins
MW	Molecular weight
NAD	Nicotinamide adenine dinucleotide
NF-kB	Nuclear factor kappa-light-chain-enhancer of activated B cells
NHANES	National Health and Nutritional Examination Survey
NO	Nitric oxide
NAFLD	Non-alcoholic Fatty Liver Disease
Nrf2	Nuclear factor erythroid 2-related factor 2
PPARγ	Peroxisomes proliferator activated receptor gamma
SIRT1	Sirtuin-1
SOD	Superoxide dismutase
STAT 3	Signal transducer and activator of transcription 3
TGs	Triglycerides
TNF	Tumour Necrosis factor
TXNRD	Thioredoxin reductase
VLDL	Very low-density lipoproteins
VO	Visceral obesity
WHO	World Health Organization
